# Supplementary material for: The interactome of intact mitochondria by cross-linking mass spectrometry provides evidence for coexisting respiratory supercomplexes
Source: Mol Cell Proteomics. 2017 Dec 8;17(2):216–32. doi: 10.1074/mcp.RA117.000470 (PMC5795388; doi:10.1074/mcp.RA117.000470)
Supplement: Supplemental Data [file supp_RA117.000470_133922_0_supp_23978_fzffwf.zip › spectra_annotation/mito_DR_spectra_annotation/349-1-4-1-8-1.pdf]

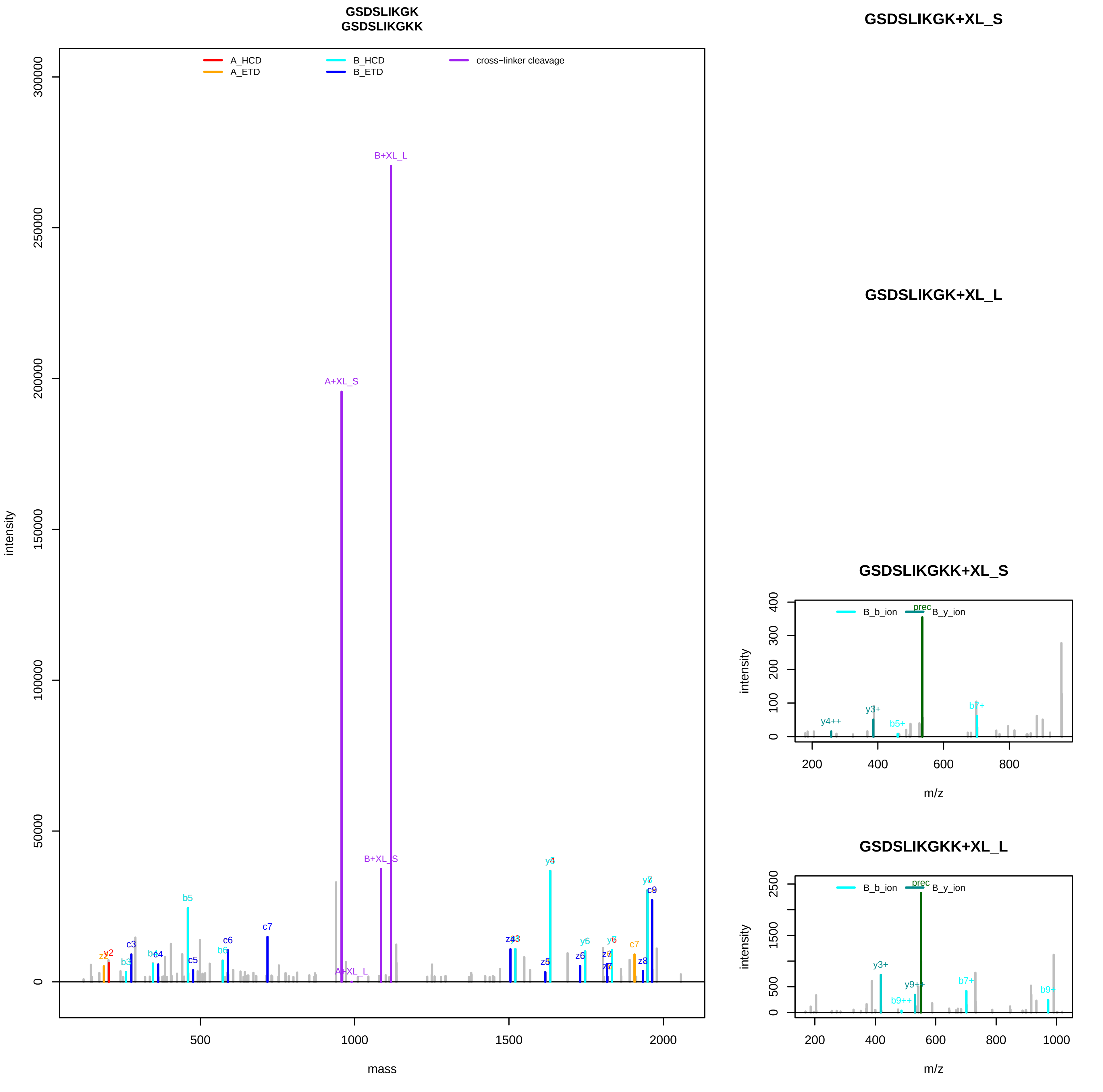
GSDSLIKGK
GSDSLIKGKK
A_HCD
A_ETD
B_HCD
B_ETD
cross−linker cleavage
B+XL_L
A+XL_S
B+XL_S
A+XL_L
intensity
mass
300000
250000
200000
150000
100000
50000
0
500
1000
1500
2000
b3
c3
c4
b5
c5
b6
c6
c7
z5
z6
y5
c9
z8
GSDSLIKGK+XL_S
GSDSLIKGK+XL_L
GSDSLIKGKK+XL_S
B_b_ion
B_y_ion
prec
y4++
y3+
b5+
b7+
intensity
m/z
400
300
200
100
0
200
400
600
800
GSDSLIKGKK+XL_L
B_b_ion
B_y_ion
prec
y3+
y9++
b9++
b7+
b9+
intensity
m/z
2500
1500
500
0
200
400
600
800
1000
